# Supplementary material for: Nontoxic and Naturally Occurring Active Compounds as Potential Inhibitors of Biological Targets in Liriomyza trifolii
Source: Int J Mol Sci. 2022 Oct 24;23(21):12791. doi: 10.3390/ijms232112791 (PMC9657120; doi:10.3390/ijms232112791)
Supplement: Supplementary file 1 [file ijms-23-12791-s001.zip › ijms-1898175-supplementary.pdf]

PROCHECK

(a)

Ramachandran Plot

saves

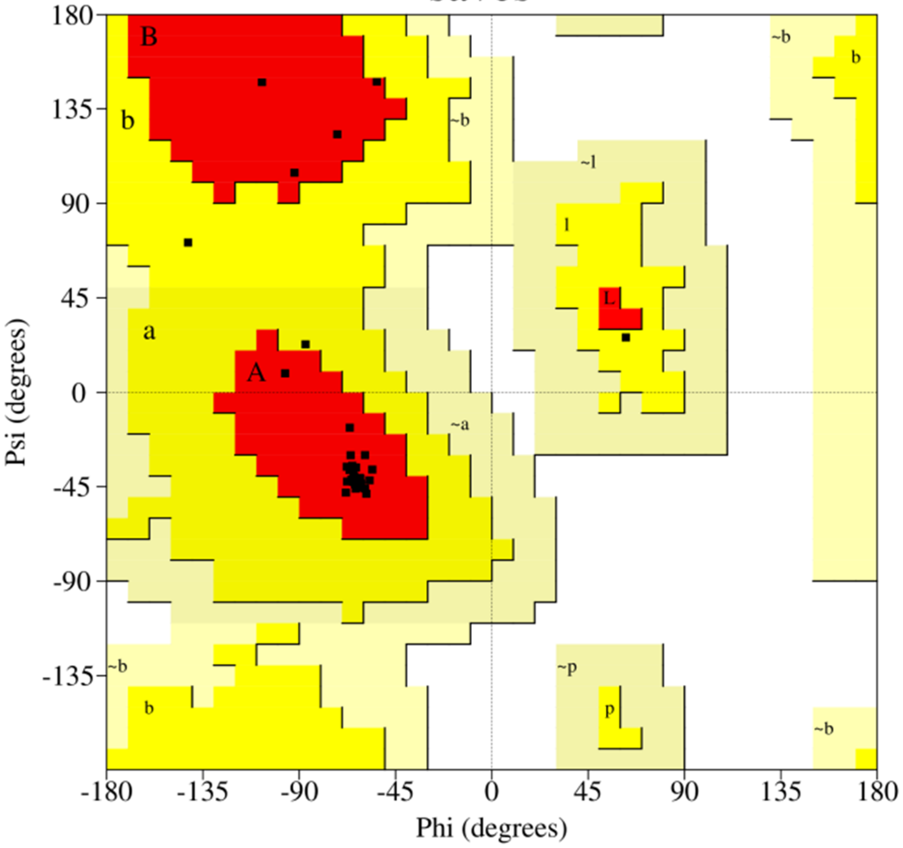

Plot statistics

Residues in most favoured regions [A,B,L]	36	92.3%
Residues in additional allowed regions [a,b,l,p]	3	7.7%
Residues in generously allowed regions [~a,~b,~l,~p]	0	0.0%
Residues in disallowed regions	0	0.0%
	----	------
Number of non-glycine and non-proline residues	39	100.0%
Number of end-residues (excl. Gly and Pro)	2	
Number of glycine residues (shown as triangles)	1	
Number of proline residues	1	

Total number of residues	43	

Based on an analysis of 118 structures of resolution of at least 2.0 Angstroms and R-factor no greater than 20%, a good quality model would be expected to have over 90% in the most favoured regions.

saves_01.ps

PROCHECK

(b)

Ramachandran Plot

saves

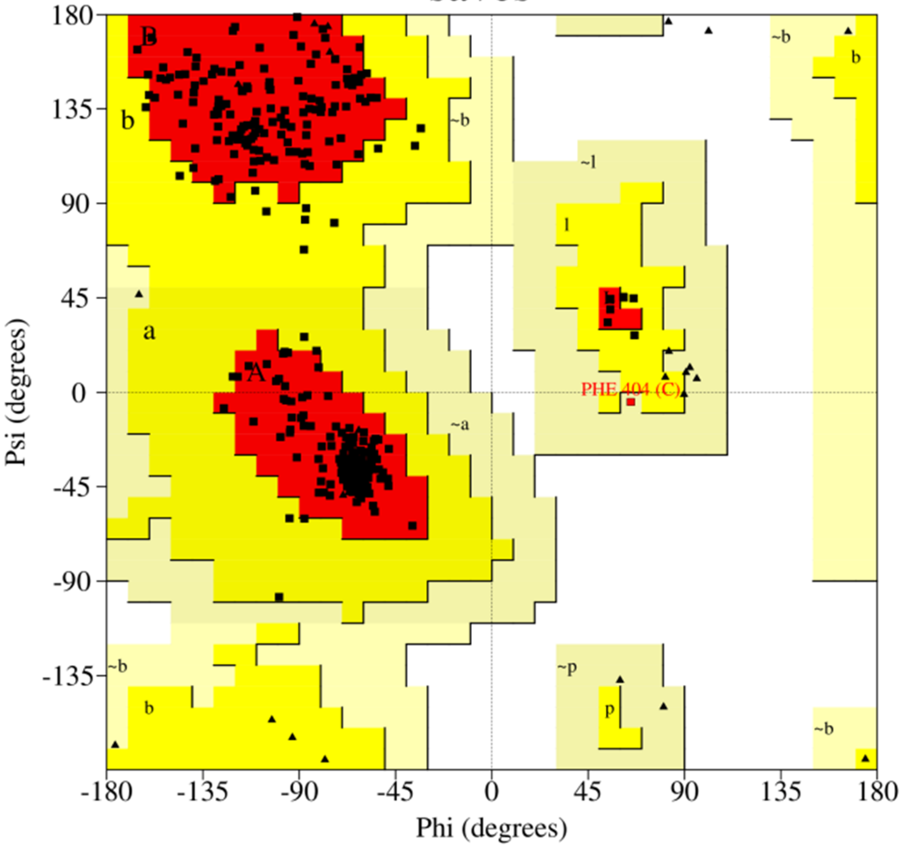

Plot statistics

Residues in most favoured regions [A,B,L]	362	94.3%
Residues in additional allowed regions [a,b,l,p]	21	5.5%
Residues in generously allowed regions [~a,~b,~l,~p]	1	0.3%
Residues in disallowed regions	0	0.0%
	----	------
Number of non-glycine and non-proline residues	384	100.0%
Number of end-residues (excl. Gly and Pro)	1	
Number of glycine residues (shown as triangles)	35	
Number of proline residues	20	

Total number of residues	440	

Based on an analysis of 118 structures of resolution of at least 2.0 Angstroms and R-factor no greater than 20%, a good quality model would be expected to have over 90% in the most favoured regions.

saves_01.ps

PROCHECK

(c)

Ramachandran Plot

saves

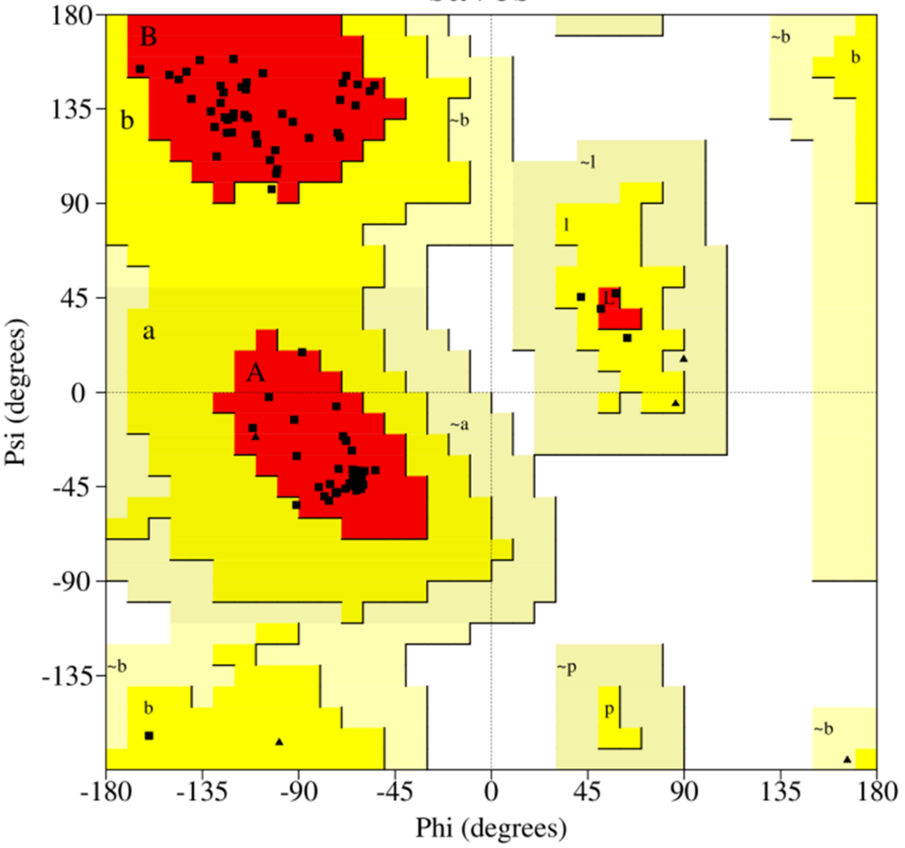

Plot statistics

Residues in most favoured regions [A,B,L]	87	94.6%
Residues in additional allowed regions [a,b,l,p]	5	5.4%
Residues in generously allowed regions [~a,~b,~l,~p]	0	0.0%
Residues in disallowed regions	0	0.0%
	----	------
Number of non-glycine and non-proline residues	92	100.0%
Number of end-residues (excl. Gly and Pro)	2	
Number of glycine residues (shown as triangles)	6	
Number of proline residues	7	

Total number of residues	107	

Based on an analysis of 118 structures of resolution of at least 2.0 Angstroms and R-factor no greater than 20%, a good quality model would be expected to have over 90% in the most favoured regions.

saves_01.ps

(d)

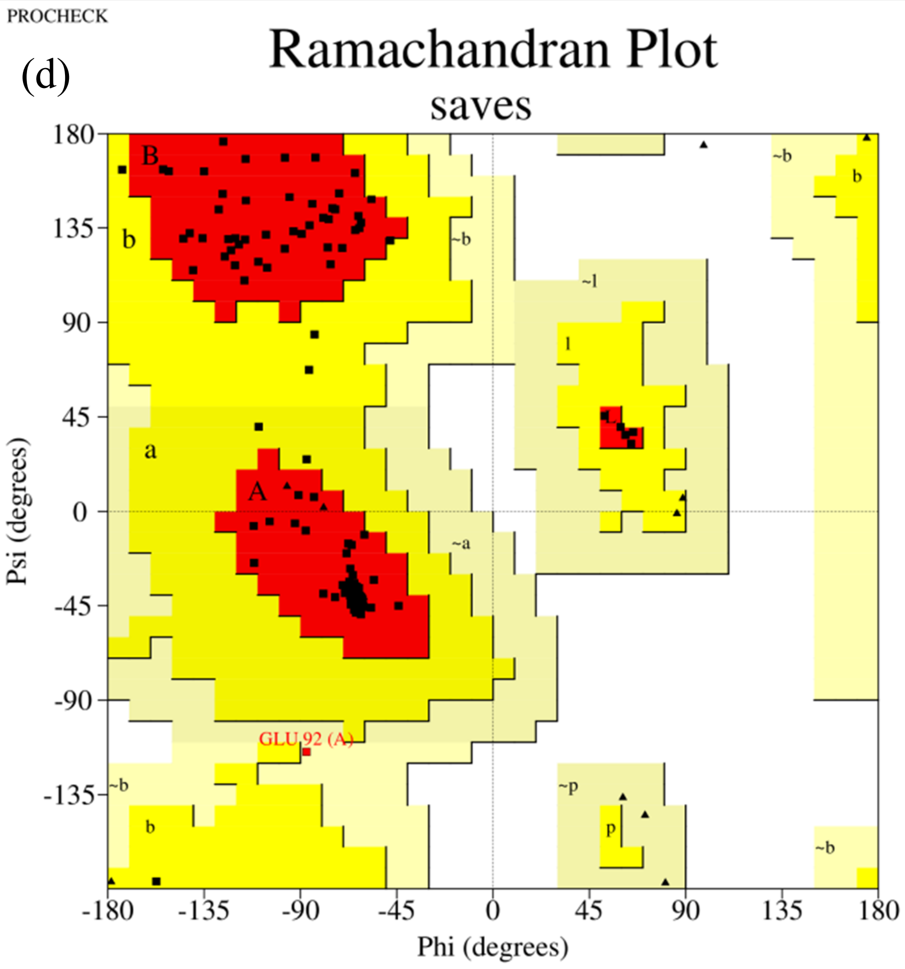

Plot statistics

Residues in most favoured regions [A,B,L]	114	94.2%
Residues in additional allowed regions [a,b,l,p]	6	5.0%
Residues in generously allowed regions [~a,~b,~l,~p]	1	0.8%
Residues in disallowed regions	0	0.0%
	----	------
Number of non-glycine and non-proline residues	121	100.0%
Number of end-residues (excl. Gly and Pro)	2	
Number of glycine residues (shown as triangles)	11	
Number of proline residues	5	

Total number of residues	139	

Based on an analysis of 118 structures of resolution of at least 2.0 Angstroms and R-factor no greater than 20%, a good quality model would be expected to have over 90% in the most favoured regions.

saves_01.ps

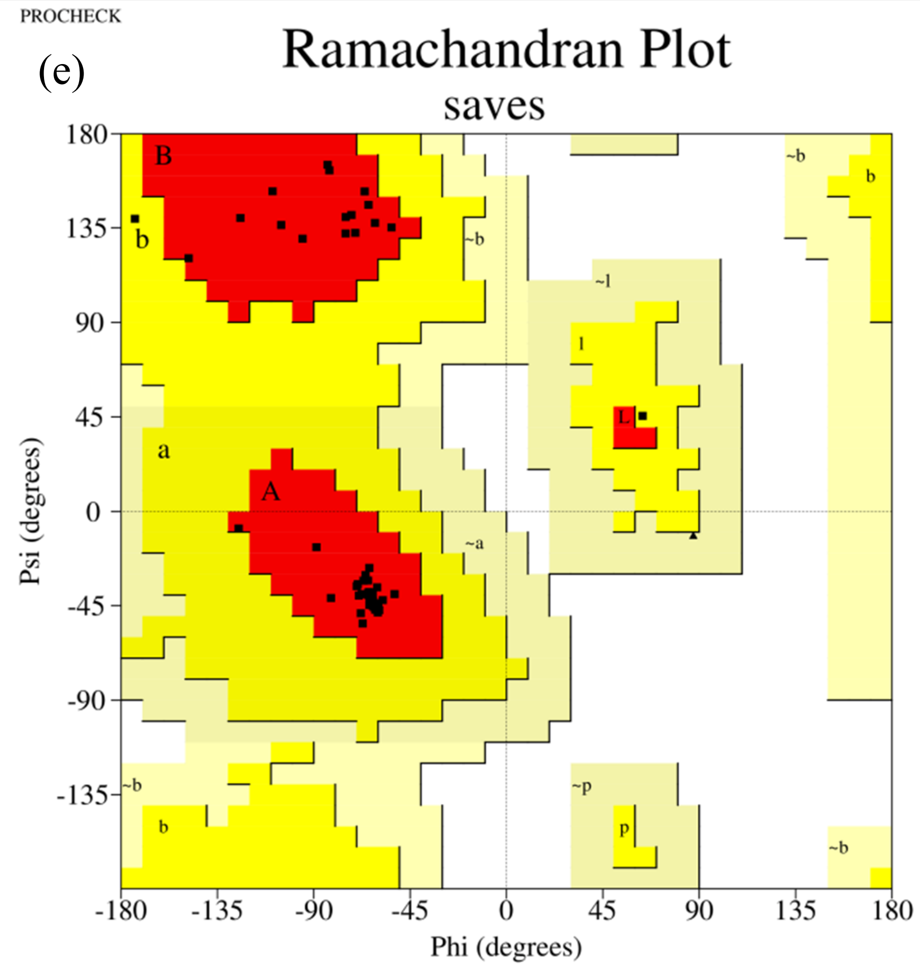

Plot statistics

Residues in most favoured regions [A,B,L]	54	96.4%
Residues in additional allowed regions [a,b,l,p]	2	3.6%
Residues in generously allowed regions [~a,~b,~l,~p]	0	0.0%
Residues in disallowed regions	0	0.0%
	----	------
Number of non-glycine and non-proline residues	56	100.0%
Number of end-residues (excl. Gly and Pro)	1	
Number of glycine residues (shown as triangles)	1	
Number of proline residues	3	

Total number of residues	61	

Based on an analysis of 118 structures of resolution of at least 2.0 Angstroms and R-factor no greater than 20%, a good quality model would be expected to have over 90% in the most favoured regions.

saves_01.ps

PROCHECK

(f)

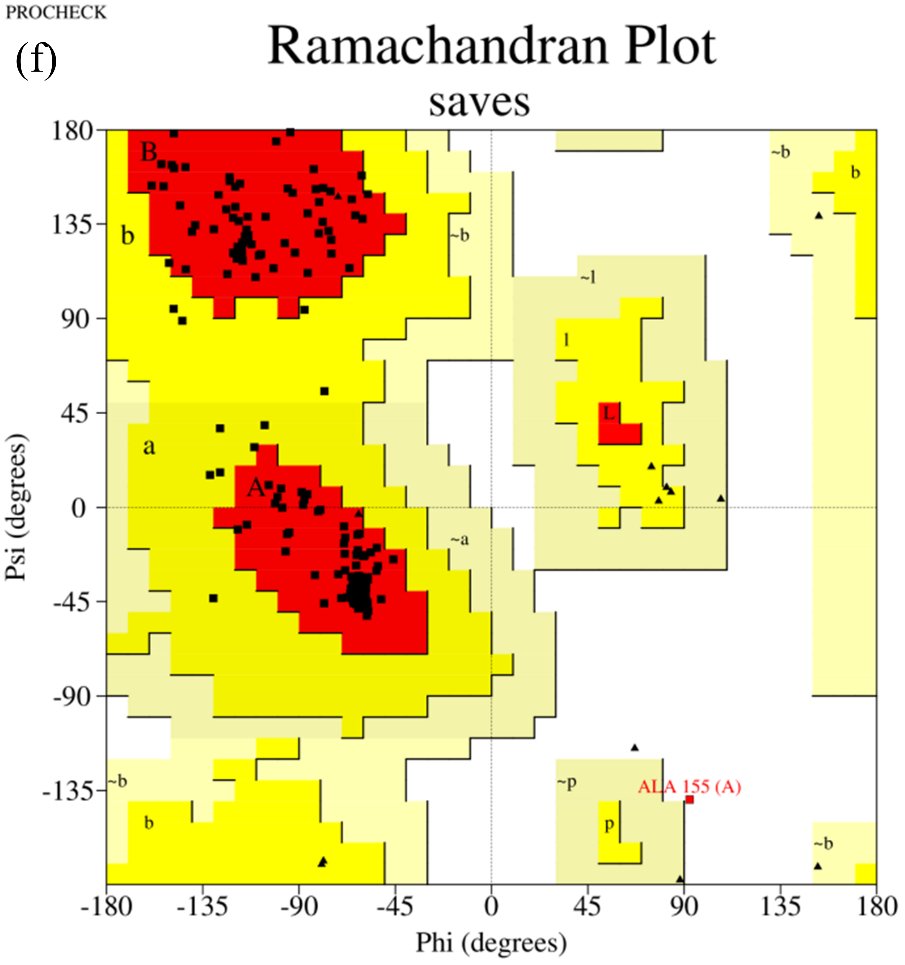

Plot statistics

Residues in most favoured regions [A,B,L]	172	93.5%
Residues in additional allowed regions [a,b,l,p]	11	6.0%
Residues in generously allowed regions [~a,~b,~l,~p]	0	0.0%
Residues in disallowed regions	1	0.5%
	----	------
Number of non-glycine and non-proline residues	184	100.0%
Number of end-residues (excl. Gly and Pro)	2	
Number of glycine residues (shown as triangles)	17	
Number of proline residues	4	

Total number of residues	207	

Based on an analysis of 118 structures of resolution of at least 2.0 Angstroms and R-factor no greater than 20%, a good quality model would be expected to have over 90% in the most favoured regions.

saves_01.ps

PROCHECK

(g)

Ramachandran Plot

saves

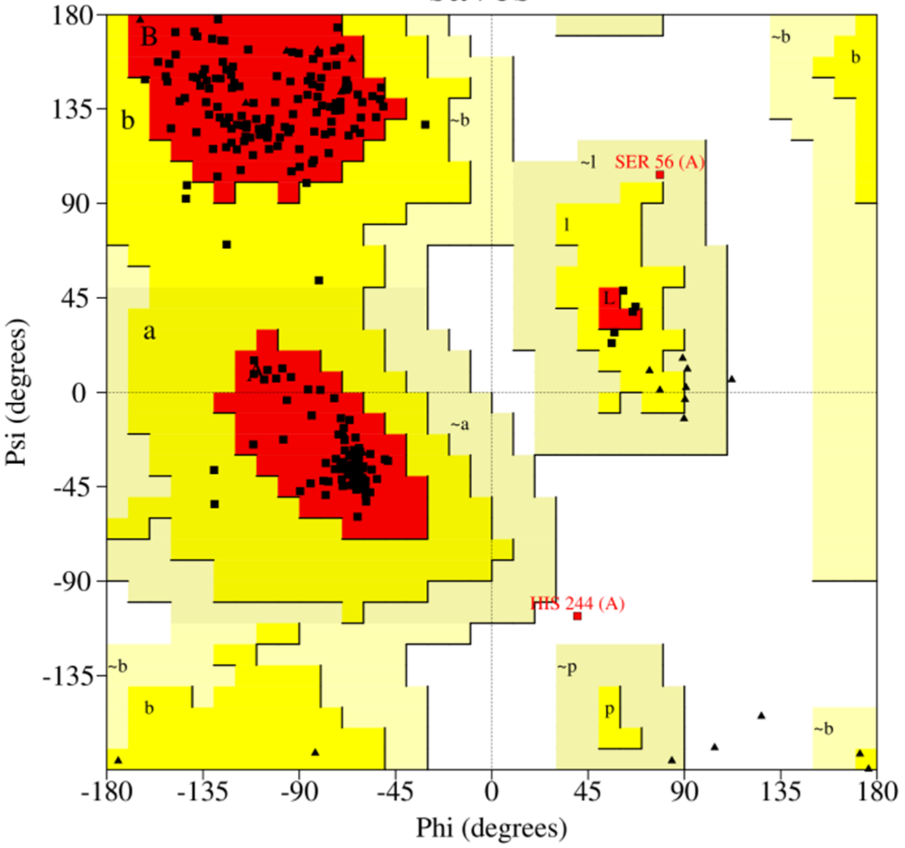

Plot statistics

Residues in most favoured regions [A,B,L]	219	94.0%
Residues in additional allowed regions [a,b,l,p]	12	5.2%
Residues in generously allowed regions [~a,~b,~l,~p]	1	0.4%
Residues in disallowed regions	1	0.4%
	----	------
Number of non-glycine and non-proline residues	233	100.0%
Number of end-residues (excl. Gly and Pro)	2	
Number of glycine residues (shown as triangles)	25	
Number of proline residues	16	

Total number of residues	276	

Based on an analysis of 118 structures of resolution of at least 2.0 Angstroms and R-factor no greater than 20%, a good quality model would be expected to have over 90% in the most favoured regions.

saves_01.ps

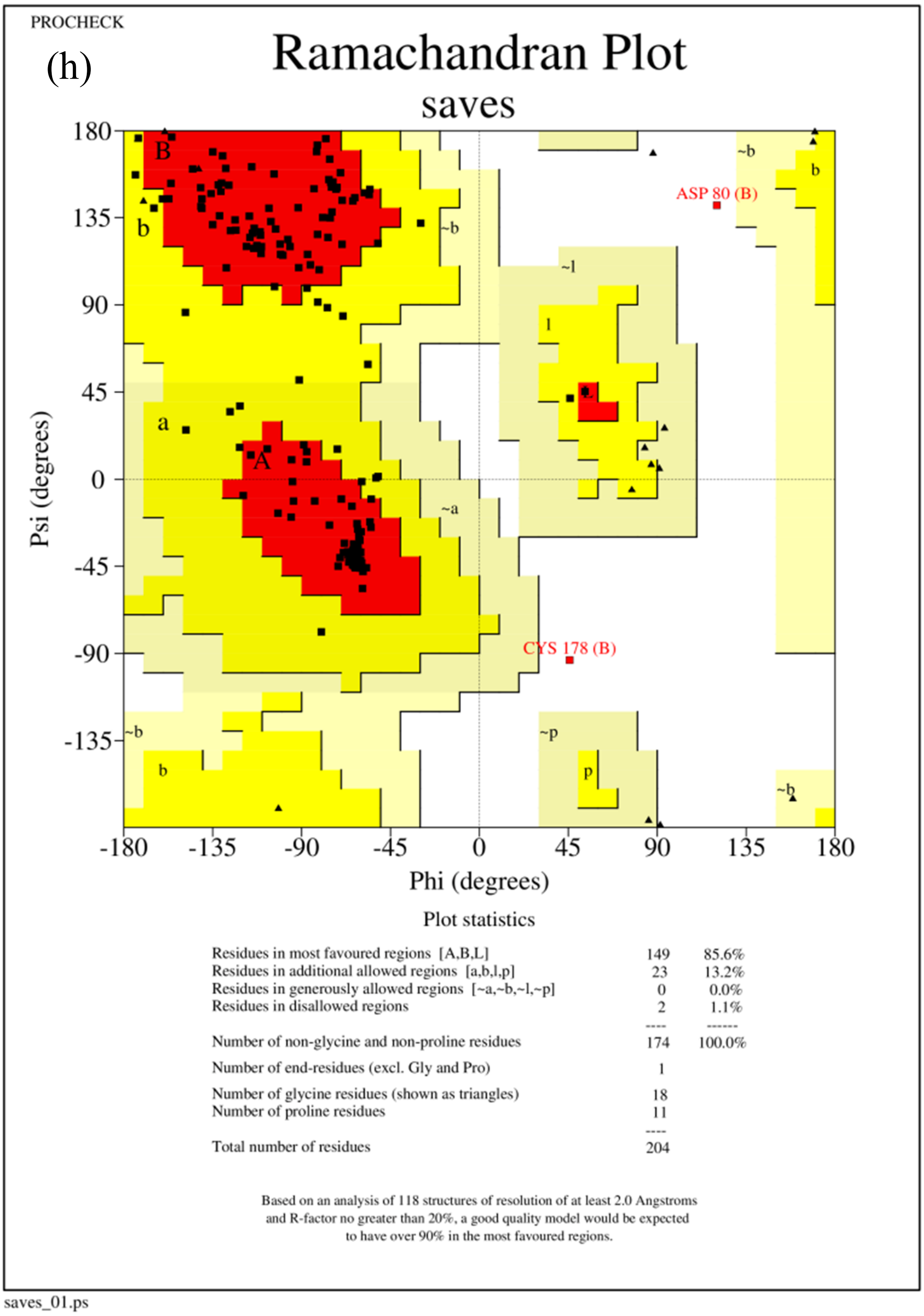

Figure S1. Ramachandran plot analysis of **a**: Acetylcholinesterase, **b**: *α*-tubulin, **c**: Actin, **d**: Argenin kinase, **e**: Histone subunit 3, **f**: Hsp90, **g**: Elongation factor and **h**: Carbamoyl phosphate synthase. *Here, the red color indicates a region that is favored energetically and structurally*, yellow region for allowed, light yellow shows generously allowed region, and white for disallowed region. Phi (Φ) and Psi (Ψ) angles determine torsion angles. Based on an analysis of the structures of resolution of at least 2.0 Å and R-factor no greater than 20%, a good quality model would be expected to have over 90% in the most favoured regions.
